# Garlic Extract Diallyl Sulfide (DAS) Activates Nuclear Receptor CAR to Induce the *Sult1e1* Gene in Mouse Liver

**DOI:** 10.1371/journal.pone.0021229

**Published:** 2011-06-15

**Authors:** Tatsuya Sueyoshi, William D. Green, Kellie Vinal, Tyler S. Woodrum, Rick Moore, Masahiko Negishi

**Affiliations:** Pharmacogenetics Section, Laboratory of Reproductive and Developmental Toxicology, National Institute of Environmental Health Sciences, National Institutes of Health, Research Triangle Park, North Carolina, United States of America; Calypte Biomedical Corporation, United States of America

## Abstract

Constituent chemicals in garlic extract are known to induce phase I and phase II enzymes in rodent livers. Here we have utilized *Car* ^+/+^ and *Car* ^−/−^ mice to demonstrate that the nuclear xenobiotic receptor CAR regulated the induction of the estrogen sulfotransferase *Sult1e1* gene by diallyl sulfide (DAS) treatment in mouse liver. DAS treatment caused CAR accumulation in the nucleus, resulting in a remarkable increase of SULT1E1 mRNA (3,200 fold) and protein in the livers of *Car* ^+/+^ females but not of *Car* ^−/−^ female mice. DAS also induced other CAR-regulated genes such as *Cyp2b10*, *Cyp3a11* and *Gadd45β*. Compared with the rapid increase of these mRNA levels, which began as early as 6 hourrs after DAS treatment, the levels of SULT1E1 mRNA began increasing after 24 hours. This slow response to DAS suggested that CAR required an additional factor to activate the *Sult1e1* gene or that this activation was indirect. Despite the remarkable induction of SULT1E1, there was no decrease in the serum levels of endogenous E2 or increase of estrone sulfate while the clearance of exogenously administrated E2 was accelerated in DAS treated mice.

## Introduction

Garlic has been consumed for food and medicinal purposes worldwide for thousands of years. Garlic's beneficial effects on human health have been known for a long time. Currently, the garlic plant itself as well as its numerous extracts are commercially available as dietary supplements. Epidemiological studies suggest garlic consumption has preventive effects for some types of gastric cancer [1]–[3]. The pharmacological basis for these preventive effects of garlic consumption have been suggested to be due to the inductions of various hepatic drug-metabolizing phase I and phase II enzymes [4]–[8]. Many of these enzymes are also induced by various CAR activators such as phenobarbital, phenytoin and TCPOBOP [9]–[13]. Whether CAR is essential for garlic extracts to induce these enzymes, however, has not been well established.

CAR, an orphan member of the nuclear steroid hormone receptor superfamily, was first found to trans-activate the CYP2B genes following phenobarbital and TCPOBOP treatment [14], [15]. Subsequent investigations have characterized CAR as a xenobiotic-activated nuclear receptor, extended CAR activators to include numerous xenobiotics and endobiotics, and increased the large number of CAR-regulated genes [9], [16]–[22]. As a result, the role of CAR has also widened from drug metabolism to various other hepatic functions such as energy homeostasis [23]–[26]. Upon xenobiotic activation, CAR is also known to become a risk factor that mediates the development of liver injuries such as steatosis, and hepatocellular carcinoma [27]–[30]. If, in fact, garlic activates CAR, this activation may have a wide range of both physiological and pathophysiological consequences.

Estrogen sulfotransferase (EST) encoded by *Sult1e1* and *SULT1E1* genes in mice and humans, respectively, is a phase II drug metabolizing enzyme localized in the cytoplasm [31], [32]. Biochemical and enzyme kinetic analysis have shown that ESTs in mice and humans catalyze the conjugation of a sulfate group at the 3-hydroxyl position of estrogens at their physiological concentrations [33]–[35]. Since sulfated estrogens do not bind the estrogen receptor as effectively, EST is considered to be a major factor for estrogen inactivation and metabolism [31], [32]. In fact, gene targeting disruption of *Sult1e1* caused elevated estrogen levels systemically and in the amniotic fluid in pregnant mice [36]. On the other hand, sulfated estrogen circulating in the blood can be converted to estrogen at the target tissues by steroid sulfatase. Thus estrogen sulfotransferase works not only for elimination of estrogen but also to produce precursors for active estrogen in a form that is more soluble and easy to deliver to the peripheral tissues [31], [32].

Here we have found that DAS, a major component of garlic extract, induced the estrogen sulfotransfease *Sult1e1* gene in mouse livers and we have investigated the role of CAR in this induction using *Car* ^−/−^ mice. In addition, serum estrogen levels and estrogen clearance were measured to determine whether SULT1E1 induction by DAS modulated the plasma levels of estrogens.

## Results

### *Sult1e1* gene induction in mouse liver

**Figure 1:**
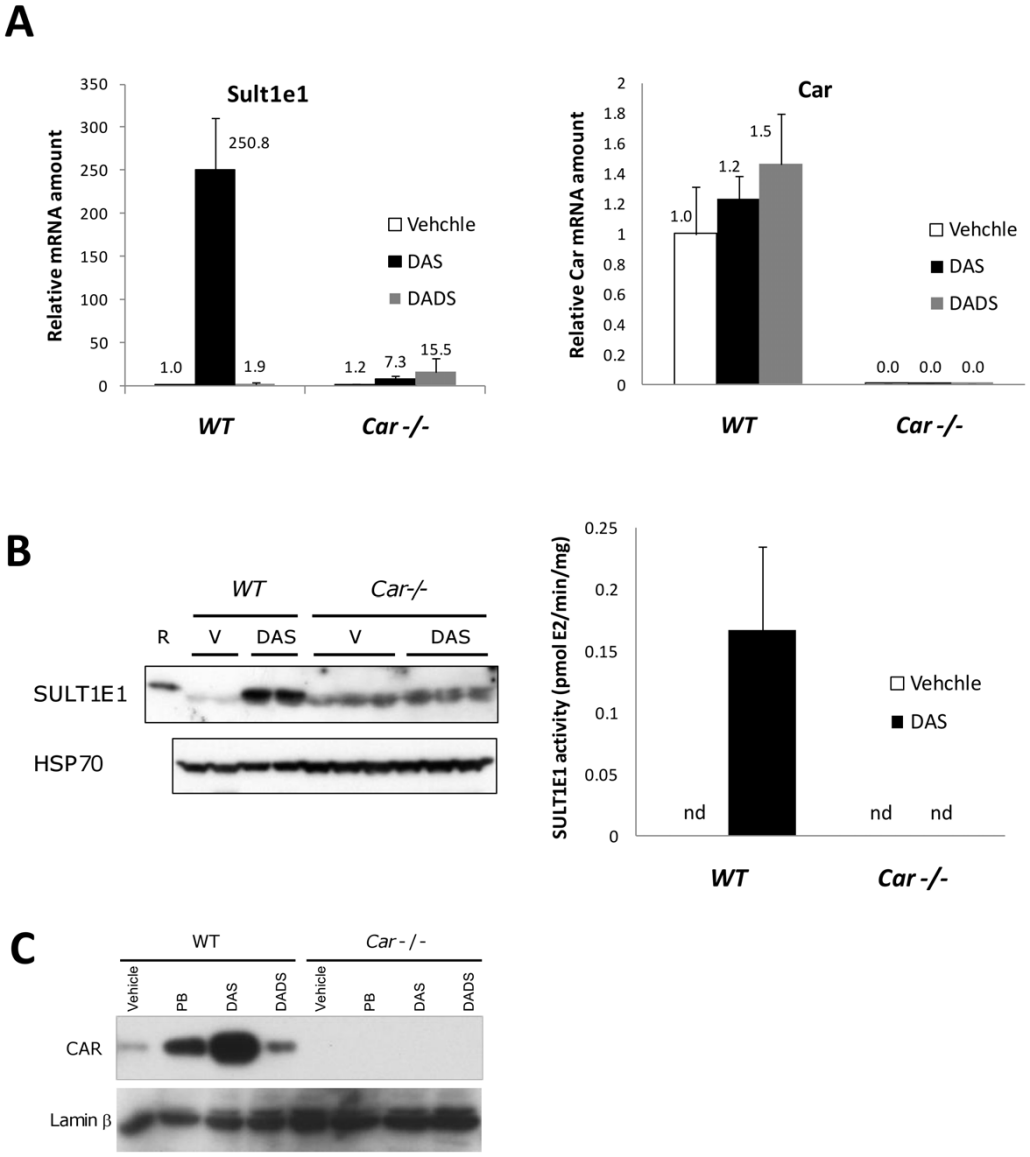
CAR dependent *Sult1e1* gene induction. (A) Wild type and *Car* null mice were given DAS (80 mg/100 g body weight) or DADS (8 mg/100 g body weight) by gastric gavage. 24 hrs after chemical administration, *Sult1e1* and *Car* gene expression was analyzed by quantitative PCR as described in the methods section. The relative mRNA amount of each gene was calculated so that wild type vehicle treated samples have one unit of expression. (B) Left panel; Liver cytosol extracts from the same mice used in (A) were prepared and SULT1E1 protein content was analyzed by western blotting. Recombinant mouse SULT1E1 protein was used as positive control and indicated with R, while V represents vehicle treatment. Anti Hsp70 western blotting is shown as a loading control. Right panel; Estrogen sulfotransferase activities were measured for liver extracts from the same mice used in (A). n.d. stands for no activity detected. (C) Liver nuclear extracts from the same mice used in (A) were prepared and CAR protein content was analyzed by western blotting. Anti Laminβ western blotting is shown as a loading control.

*Sutl1e1* gene expression was analyzed in wild type and *Car* null female mice treated with DAS and DADS as described in the methods section. DAS dramatically induced (250-fold) the gene expression at 24 hrs after DAS gastric administration (Fig. 1a). In contrast, only a marginal induction (7.3 fold) of the same gene was observed in *Car* null mice. Conversely, diallyl disulfide (DADS) induces this gene 1.9 fold in wild type mice and 15.5 fold in *Car* null mice, suggesting that this chemical does not activate CAR for *Sult1e1* gene induction. In agreement with mRNA expression, SULT1E1 protein in liver cytosol was induced by DAS as shown in the western blot in Fig. 1b, left panel, while in *Car* null mice livers, DAS had negligible effects on the protein content. The protein detected by the anti-SULT1E1 antibody co-migrated with recombinant mouse SULT1E1 protein on the SDS-PAGE gel. The estrogen sulfotransferase activity of the liver extracts used in Fig. 1a is shown in Fig. 1b right panel. These results were well consistent with the detected mRNA levels and protein levels in Fig. 1a and Fig. 1b. Next, we analyzed changes in CAR protein content in the nucleus of liver cells following DAS treatment, since CAR is known to migrate into the nucleus from the cytoplasm upon its activation. Thus, we analyzed CAR protein levels in mouse liver nuclear extracts before and after DAS administration. We observed that levels of CAR protein in the nucleus were increased by DAS and DADS administration in wild type animals, while no CAR protein was detected with the same antibody in *Car* null mice nuclear extracts. The effect of DAS was so dramatic that the CAR protein content in the nucleus was much higher than that of PB treated mice. The reason why DADS activates CAR for nuclear translocation but not for Sult1e1 gene activation may be an interesting topic for research in the future.

### Gene inductions in DAS treated wild type and *Car* null mice

**Figure 2:**
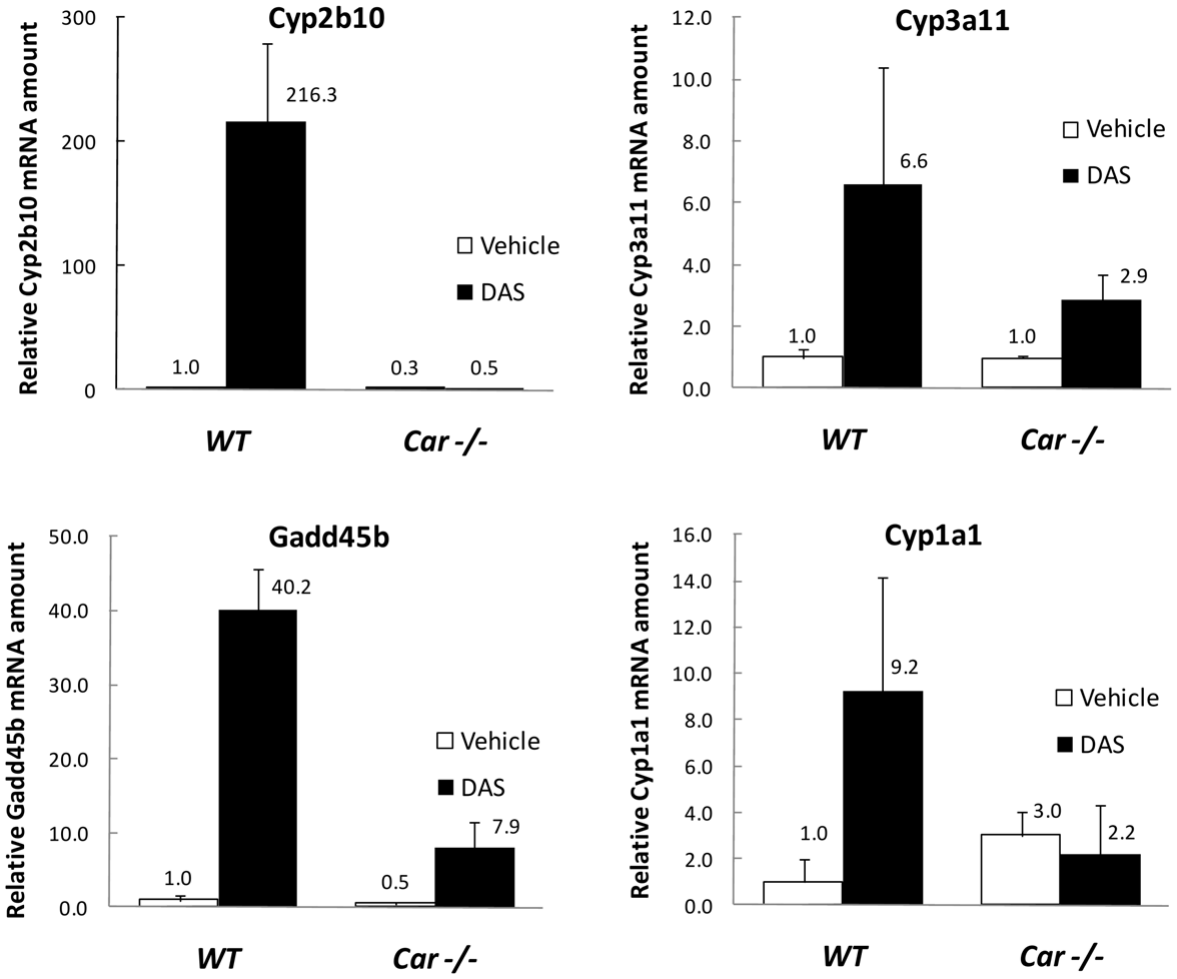
Gene activation by DAS. The expression of *Cyp2b10*, *Cyp3a11*, *Gadd45b* and *Cyp1a1* genes was analyzed in DAS treated and vehicle treated mice livers. The same RNA samples treated with vehicle or DAS from wild type and *Car* null mice liver used in FIG. 1A were analyzed to determine the expression of these genes.

In DAS treated mouse liver, other xenobiotic metabolizing enzymes were also induced as shown in Fig. 2. The most extensively analyzed CAR dependent gene in mouse liver, *Cyp2b10*, was markedly induced almost to the same extent (216 fold) as *Sult1e1* while no induction of this gene was observed in *Car* null mice. In the case of *Cyp3a11*and *Gadd45b*, significant induction was observed in wild type mice while less induction was found in *Car* null mice. For *Cyp1a1*, no gene induction was observed in *Car* null mice while significant induction was observed in wild type mice, suggesting that CAR plays a critical role in the induction of *Cyp1a1* by DAS. These observations suggest that there may be regulatory elements in the mouse *Cyp1a1* gene that can be activated by CAR, as in the case of the human *CYP1A1* gene [37].

### Time course *Sult1e1* gene activation by DAS

**Figure 3:**
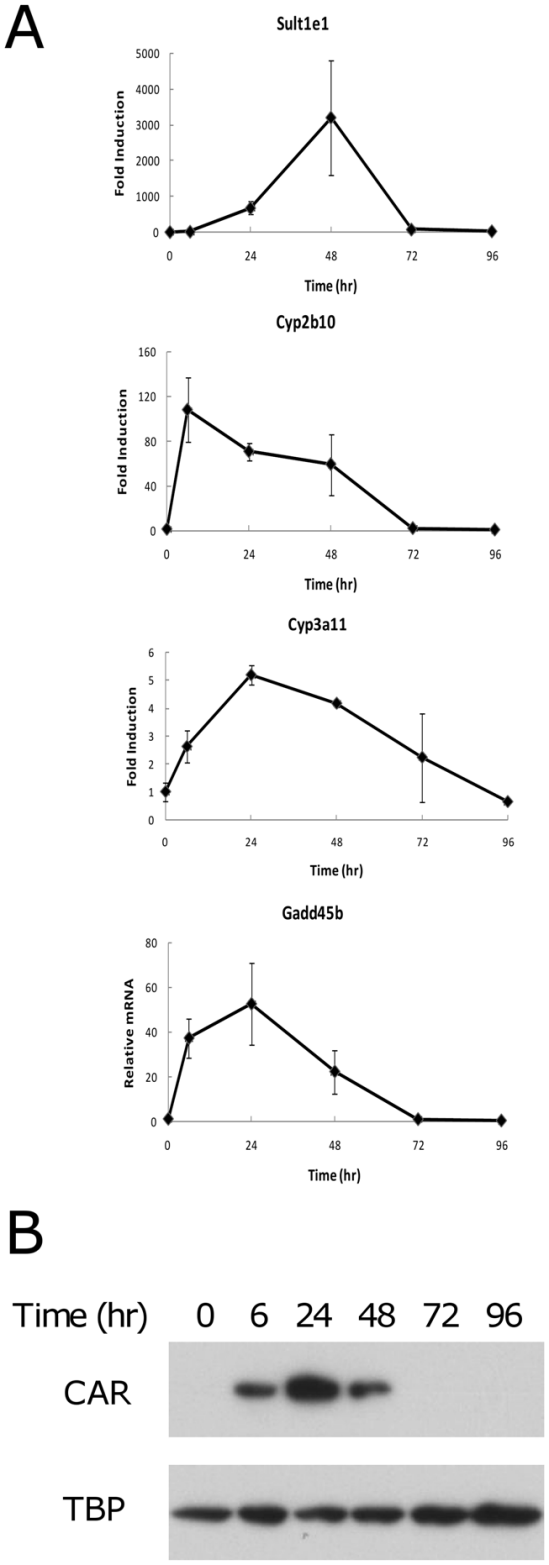
Gene induction time course. (A) Wild type and *Car* null mice were given DAS (80 mg/100 g body weight) by gastric gavage. B. Following 6, 24, 48, 72, and 96 hrs after chemical administration, the expression of *Sult1e1*, *Cyp2b10*, *Cyp3a11*, and *Gadd45b* genes was analyzed by quantitative PCR as described in the methods section. The relative mRNA amount of each gene was calculated so that wild type vehicle treated samples have one unit of expression. (B) Liver nuclear extracts from DAS treated mice at indicated time after the chemical administration were prepared and CAR protein content was analyzed by western blotting. Anti TATA binding protein (TBP) western blotting is shown as a loading control.

Next, we analyzed the time course of the increase of gene expression after DAS treatment. *Sult1e1* and *Cyp2b10* gene expression was analyzed at 6, 24, 48, 72, and 96 hrs after DAS gavage (Fig. 3a). *Cyp2b10* was very quickly induced compared to *Sult1e1*. At 6 hrs, *Cyp2b10* induction was already at the maximum while *Sult1e1* induction was very low at this point. *Sult1e1* expression levels increased up to 48 hrs after DAS treatment to over 3000 fold and then decreased to an almost basal level at 72 hrs. In contrast, *Cyp2b10* expression was gradually decreased from its 6 hr maximum (110 fold) to 60 fold at 48 hrs, and returned to basal levels at 72 hrs. In the meantime, we observed strong CAR accumulation in the nucleus at 6, 24, and 48 hrs while no CAR protein existed in the nucleus at 72 or 96 hrs (Fig. 3b). This dramatic difference in the time course for induction of these two genes suggests that partially independent mechanisms are playing roles for the induction of each gene. The time courses for induction of the *Cyp3a11* and *Gadd45b* genes were intermediate to those of *Sult1e1* and *Cyp2b10*, with maximum expression at 24 hrs and decreasing gradually to the basal level at 72 hr or 96 hrs.

### Serum estrogen levels in DAS treated mice

**Figure 4:**
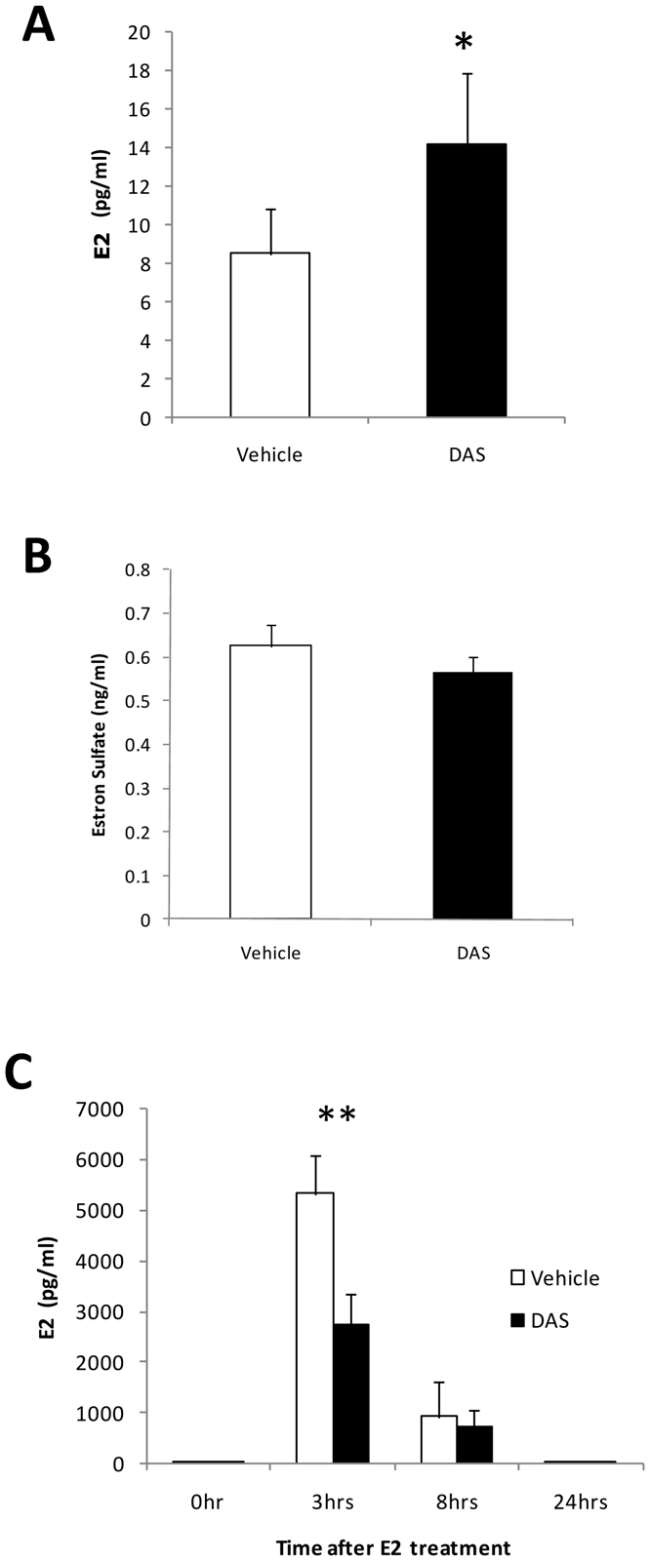
The effect of DAS treatment on serum estrogen. (A) E2 levels in sera from 4 week old mice treated with vehicle or DAS for 48 hrs are shown. * p<0.05 for vehicle-injected group versus DAS-injected group. (B) Estrone sulfate levels in sera from 8 week old female mice treated with vehicle or DAS for 48 hrs are shown. (C) The clearance of exogenously administered E2 from mice sera was analyzed using DAS treated and vehicle treated ovariectomized mice. Ovariectomized mice were given DAS via gavage and E2 (0.5 mg/kg) was injected subcutaneously 42 hrs after the DAS administration. The E2 content in serum was determined by radioimmunoassay and plotted against time after E2 treatment. **, p<0.01 for vehicle-injected group versus DAS-injected group at 3 hrs after E2 treatement.

We analyzed serum estrogen levels in DAS-treated mice. Surprisingly there was slight increase in estrogen levels in the serum of 4 week old mice after DAS treatment (Fig. 4a). Estrone sulfate levels in serum did not change with the same treatment (Fig. 4b). Thus we measured the clearance of exogenously administrated estrogen in ovariectomized mice with or without DAS pretreatment for 42 hrs to allow for maximal induction of the *Sult1e1* gene. Then, the mice were administrated with estrogen (0.5 mg/kg) and estrogen levels in serum were determined at 3, 8, and 24 hrs after the administration (Fig. 4c). E2 was shown to be very rapidly cleared from the serum and at 24 hrs following administration, E2 levels in DAS treated and control mice were the same as the basal levels found in non E2 administered mice. At 3 hrs after E2 administration, the E2 levels in DAS treated mice were significantly lower than those of non treated mice, while at 8 hrs no difference was observed.

## Discussion

Naturally-occurring organosulfur compounds including DAS found in garlic and onions can induce diverse phase I and phase II enzymes in mice and rats [4], [5], [8]. Many of these enzyme inductions seem to be mediated by CAR activation [6], [7], [38]. These gene inductions affect endogenous and exogenous chemical metabolism and the gene expression and metabolism change brought by garlic consumption should explain garlic's beneficial effect on the body. Epidemiological studies established garlic's preventive effects for colorectal and stomach cancer [1]–[3]. Cancer incidents in other sites (head and neck, lung, breast, and prostate) are also suggested to be reduced with garlic consumption. Furthermore, *Sult1e1* polymorphisms are shown to be a risk factor for breast and endometrial cancers [39]–[41]. Thus, where *CAR* and *SULT1E1* genes are co-expressing, garlic may affect cancer formation and development by affecting *SULT1E1* gene expression. However, there are no overall clear connections between the animal model studies and human epidemiological studies established so far. One group has shown that organosulfur compounds can reduce aflatoxin B1 genotoxicity in rats by inducing phase I and phase II enzymes that increase the metabolism of aflatoxin B1 [4]. Since garlic extract can affect the expression of diverse genes thus resulting in many changes in biological processes, much additional work is necessary using human tissue derived model systems in order to establish the relevance of animal model findings on human health.

In this report we observed a very strong induction of the *Sult1e1* gene in the mouse liver by gastrically injected DAS. CAR is a dominant factor in the induction of this gene, since only a marginal induction of this gene was observed in *Car* null mice. Recently, mouse *Sult1e1* was shown to be induced by many chemicals that activate CAR [11]. Among these chemicals, TCPOBOP, a specific mouse CAR agonist, showed more potent induction in female mice than in males [11]. We are using female mice in this report and we observed slightly less *Sult1e1* induction in male mice by DAS treatment (data not shown).

The *Sult1e1* gene induction time course was found to be unique as shown in Fig. 3. Other canonical genes induced by CAR activation had their highest expressions between 6–24 hrs. In contrast, Sult1e1 gene induction was optimum at 48 hrs after chemical administration. This observation suggests secondary factors which are activated by CAR are involved in *Sult1e1* gene induction. Recently, the paracrine regulatory mechanism of the *SULT1E1* gene was characterized in HepG2 cells co-cultured with MMNK-1 cholangiocytes [42], [43]. When HepG2 cells were co-cultured with CFTR siRNA treated MMNK-1 cells in a Transwell system, an unidentified factor secreted from MMNK-1 cells that is permeable through the Transwell membrane activated *SULT1E1* gene expression in HepG2 cells. Whether such a paracrine mechanism is involved in *Sult1e1* induction by DAS, thus causing a slow gene induction in contrast to other CAR responsive genes, remains unanswered at this point. Another recent report suggests that RORα suppresses *Sult1e1* expression in mice [44]. The broad spectrum of RORα and RORγ in the regulation of phase I and phase II genes implies that these receptors have crosstalk with CAR. Whether such a crosstalk plays a role or not in *Sult1e1* gene induction by DAS will be an interesting research target.

A striking finding of this report is that the strong *Sult1e1* gene induction did not affect the endogenous E2 level while improved E2 elimination was observed when the hormone was exogenously administered to ovariectomized mice. A recent report demonstrated that E2 decreased in mice when the *Sult1e1* gene was induced in mouse livers by glucocorticoid [45]. They suggested that one of the mechanisms by which glucocorticoid attenuates E2's biological effect is augmenting *Sult1e1* expression in the liver so that conversion of serum E2 to its sulfated form for eventual secretion from the body is accelerated. In contrast, although DAS treated mice have robust *Sult1e1* induction (around 3200 fold), endogenous E2 was not affected although exogenous E2 clearance was faster as shown in Fig. 4. These results suggest that *Sult1e1* in the liver may not be the dominant regulator for the E2 levels in the mice of our experimental systems. One additional point is that the endogenous E2 levels were analyzed in 4 week old female mice while 8 week old ovariectomized mice were used for E2 elimination experiment. Although the *Sult1e1* gene in liver was induced in both cases (data not shown), we don't know if this age difference can affect the whole body response to DAS. Moreover, DAS may have effect on estradiol synthesis pathways, estradiol turnover or expression/activity of SULT1E1 in the tissues other than liver. Further detailed pharmacokinetical, pharmacogenetical, and biochemical studies are necessary to determine why this strong induction of *Sult1e1* does not alter serum E2 levels.

## Materials and Methods

### Animals

*Car* null mice with C3H/HeNCrlBR (C3H) genetic background were established as previously described [28]. Mice were kept in 12∶12h dark/light cycle and fed with a standard solid diet *ad libitum*. C3H wild type and *Car* null female mice received DAS (80 mg/100 g body weight) and DADS (8 mg/100 g) via gavage using corn oil as the vehicle. Phenobarbital (1 mg/100 g) dissolved in PBS was administrated intraperitoneally. Mice were sacrificed at specified hours after the administration of xenobiotics for liver RNA or serum preparation. All animal procedures were approved by the Animal Ethics Committee at NIEHS, NIH (Permit Number: ASP 00-19).

### Chemicals

DAS (allyl sulfide, 97%) and DADS (allyl disulfide, 80%) were purchased from Sigma.

### Real Time RT PCR

Total mouse liver (three C3H female mice for each data point) RNA was isolated with Trizol reagent (Invitrogen) and reverse transcription was performed using High Capacity cDNA Archive kits for the RNA. Quantitative real-time PCR was carried out with the 7900HT Fast Real Time PCR System (Applied Biosystems). For Nr1i3, FAM-CCATGACAGCTATGCTAA-MGB probe was used with 5′-CCTGCTGCCTAAGGGAAACAG-3- and 5′-TCTTCACTGGCCATGGTTTCTA-3′ primers. For *Sult1e1*, *Cyp2b10*, *Cyp3a11*, *Gadd45b* and *Cyp1a1*, pre-synthesized probes from ABI, Mm00499178_m1, Mm00456591_m1, Mm00731567, Mm00435123_m1, and Mm00487218_m1 were used, respectively. For normalization of gene expressions among individual mice, *Gapdh* gene expressions determined with TaqMan rodent glyceraldehyde-3-phosphate dehydrogenase control reagent (Applied Biosystems) were utilized.

### Western blotting

Mouse liver cytosol and nuclear extracts from C3H female mice were prepared as described previously [46]. Individual mouse liver extracts and pooled nuclear extracts from 3 mice were used for western blotting. The protein concentration of extracts was determined using the Bio-Rad Protein Assay reagent. Thirty and 100 µg protein samples for nuclear extracts and cytosol extracts respectively were separated on a SDS-PAGE gel and transferred to PVDF membranes. Anti SULT1E1 and recombinant mouse SULT1E1 were described in the previous report [47]. Anti CAR monoclonal antibody developed by Perseus Proteomics (Tokyo, Japan) was purchased from R&D Systems (Minneapolis, MN). Anti HSP70 and anti lamin β were from BD Biosciences (San Jose, CA) and Santa Cruz Biotechnology (Santa Cruz, CA), respectively.

### Estrogen sulfotransferase activity

Estrogen sulfotransferase activities of liver extracts were determined as described previously [47]. Briefly, liver extracts were incubated with 2 µM ^3^H labeled estradiol and 100 µM 3′-Phosphoadenosine-5′-phosphosulfate in 200 mM Tris-acetate (pH 8.0) buffer containing 10 mM magnesium acetate. The samples were incubated at 37°C for 30 min and extracted with dichloromethane. The ^3^H label in the aqueous phase was counted to measure sulfated estradiol.

### Serum estrogen

Ovariectomized or non-operated female C3H mice (7weeks old) and female C3H mice (4 weeks old) were obtained from Jackson Laboratory (Bar Harbor, ME). For E2 clearance analysis, ovariectomized mice were treated with DAS or corn oil (vehicle) via gavage. Forty two hours after DAS treatment, beta E2 (0.5 mg/kg) was administrated subcutaneously. At 3, 8, and 24 hrs after E2 administration, mice were sacrificed for liver RNA preparation while blood samples were collected from the inferior vena cava. For endogenous E2 levels in 4 week old mice, mice were treated with DAS or vehicle. Serum and RNA were prepared at 48 hrs after the treatment. For estrone sulfate determination, 7 weeks old female C3H mice were utilized for DAS or vehicle treatment (48 hrs). Serum was isolated with BD Microtainer serum separator tubes (Becton, Dickinson and Co., Franklin Lakes, NJ). Serum E2 and estrone sulfate concentrations were determined with DSL-4800 Ultra-Sensitive Estradiol RIA kit and DSL-5400 Estrone Sulfate RIA kit (Beckman Coulter, Brea, CA).

### Statistical analysis

Statistical analysis was performed by Student's *t* test to compare the estradiol elimination difference between vehicle and DAS treated C3H female mice.

## References

[1] Bianchini F, Vainio H (2001). Allium vegetables and organosulfur compounds: do they help prevent cancer?. Environmental health perspectives.

[2] Fleischauer AT, Arab L (2001). Garlic and cancer: a critical review of the epidemiologic literature.. The Journal of nutrition.

[3] Ngo SN, Williams DB, Cobiac L, Head RJ (2007). Does garlic reduce risk of colorectal cancer? A systematic review.. The Journal of nutrition.

[4] Guyonnet D, Belloir C, Suschetet M, Siess MH, Le Bon AM (2002). Mechanisms of protection against aflatoxin B(1) genotoxicity in rats treated by organosulfur compounds from garlic.. Carcinogenesis.

[5] Berges R, Siess MH, Arnault I, Auger J, Kahane R (2004). Comparison of the chemopreventive efficacies of garlic powders with different alliin contents against aflatoxin B1 carcinogenicity in rats.. Carcinogenesis.

[6] Fisher CD, Augustine LM, Maher JM, Nelson DM, Slitt AL (2007). Induction of drug-metabolizing enzymes by garlic and allyl sulfide compounds via activation of constitutive androstane receptor and nuclear factor E2-related factor 2.. Drug metabolism and disposition: the biological fate of chemicals.

[7] Zhang P, Noordine ML, Cherbuy C, Vaugelade P, Pascussi JM (2006). Different activation patterns of rat xenobiotic metabolism genes by two constituents of garlic.. Carcinogenesis.

[8] Guyonnet D, Belloir C, Suschetet M, Siess MH, Le Bon AM (2001). Antimutagenic activity of organosulfur compounds from Allium is associated with phase II enzyme induction.. Mutation research.

[9] Honkakoski P, Sueyoshi T, Negishi M (2003). Drug-activated nuclear receptors CAR and PXR.. Ann Med.

[10] Urquhart BL, Tirona RG, Kim RB (2007). Nuclear receptors and the regulation of drug-metabolizing enzymes and drug transporters: implications for interindividual variability in response to drugs.. Journal of clinical pharmacology.

[11] Alnouti Y, Klaassen CD (2008). Regulation of sulfotransferase enzymes by prototypical microsomal enzyme inducers in mice.. The Journal of pharmacology and experimental therapeutics.

[12] di Masi A, De Marinis E, Ascenzi P, Marino M (2009). Nuclear receptors CAR and PXR: Molecular, functional, and biomedical aspects.. Molecular aspects of medicine.

[13] Wang H, Faucette S, Moore R, Sueyoshi T, Negishi M (2004). Human constitutive androstane receptor mediates induction of CYP2B6 gene expression by phenytoin.. J Biol Chem.

[14] Sueyoshi T, Kawamoto T, Zelko I, Honkakoski P, Negishi M (1999). The repressed nuclear receptor CAR responds to phenobarbital in activating the human CYP2B6 gene.. J Biol Chem.

[15] Honkakoski P, Zelko I, Sueyoshi T, Negishi M (1998). The nuclear orphan receptor CAR-retinoid X receptor heterodimer activates the phenobarbital-responsive enhancer module of the CYP2B gene.. Mol Cell Biol.

[16] Kawamoto T, Kakizaki S, Yoshinari K, Negishi M (2000). Estrogen activation of the nuclear orphan receptor CAR (constitutive active receptor) in induction of the mouse Cyp2b10 gene.. Mol Endocrinol.

[17] Ueda A, Hamadeh HK, Webb HK, Yamamoto Y, Sueyoshi T (2002). Diverse roles of the nuclear orphan receptor CAR in regulating hepatic genes in response to phenobarbital.. Molecular pharmacology.

[18] Wei P, Zhang J, Egan-Hafley M, Liang S, Moore DD (2000). The nuclear receptor CAR mediates specific xenobiotic induction of drug metabolism.. Nature.

[19] Kawamoto T, Sueyoshi T, Zelko I, Moore R, Washburn K (1999). Phenobarbital-responsive nuclear translocation of the receptor CAR in induction of the CYP2B gene.. Mol Cell Biol.

[20] Qatanani M, Moore DD (2005). CAR, the continuously advancing receptor, in drug metabolism and disease.. Current drug metabolism.

[21] Sugatani J, Kojima H, Ueda A, Kakizaki S, Yoshinari K (2001). The phenobarbital response enhancer module in the human bilirubin UDP-glucuronosyltransferase UGT1A1 gene and regulation by the nuclear receptor CAR.. Hepatology.

[22] Swales K, Negishi M (2004). CAR, driving into the future.. Mol Endocrinol.

[23] Kodama S, Koike C, Negishi M, Yamamoto Y (2004). Nuclear receptors CAR and PXR cross talk with FOXO1 to regulate genes that encode drug-metabolizing and gluconeogenic enzymes.. Mol Cell Biol.

[24] Konno Y, Negishi M, Kodama S (2008). The roles of nuclear receptors CAR and PXR in hepatic energy metabolism.. Drug metabolism and pharmacokinetics.

[25] Wada T, Gao J, Xie W (2009). PXR and CAR in energy metabolism.. Trends in endocrinology and metabolism: TEM.

[26] Dong B, Saha PK, Huang W, Chen W, Abu-Elheiga LA (2009). Activation of nuclear receptor CAR ameliorates diabetes and fatty liver disease.. Proceedings of the National Academy of Sciences of the United States of America.

[27] Huang W, Zhang J, Washington M, Liu J, Parant JM (2005). Xenobiotic stress induces hepatomegaly and liver tumors via the nuclear receptor constitutive androstane receptor.. Mol Endocrinol.

[28] Yamamoto Y, Moore R, Goldsworthy TL, Negishi M, Maronpot RR (2004). The orphan nuclear receptor constitutive active/androstane receptor is essential for liver tumor promotion by phenobarbital in mice.. Cancer research.

[29] Yamazaki Y, Kakizaki S, Horiguchi N, Sohara N, Sato K (2007). The role of the nuclear receptor constitutive androstane receptor in the pathogenesis of non-alcoholic steatohepatitis.. Gut.

[30] Kakizaki S, Yamazaki Y, Takizawa D, Negishi M (2008). New insights on the xenobiotic-sensing nuclear receptors in liver diseases–CAR and PXR.. Current drug metabolism.

[31] Falany CN, Lash LH (2005). Human cytosolic sulfotransferases. Properties, physiological functions, and toxicology.. Methods in pharmacology and toxicology. Drug metabolism and transport: Molecular methods and mechanism.

[32] Song WC (2001). Biochemistry and reproductive endocrinology of estrogen sulfotransferase.. Annals of the New York Academy of Sciences.

[33] Falany CN, Krasnykh V, Falany JL (1995). Bacterial expression and characterization of a cDNA for human liver estrogen sulfotransferase.. The Journal of steroid biochemistry and molecular biology.

[34] Kakuta Y, Pedersen LG, Carter CW, Negishi M, Pedersen LC (1997). Crystal structure of estrogen sulphotransferase.. Nature structural biology.

[35] Petrotchenko EV, Doerflein ME, Kakuta Y, Pedersen LC, Negishi M (1999). Substrate gating confers steroid specificity to estrogen sulfotransferase.. The Journal of biological chemistry.

[36] Tong MH, Jiang H, Liu P, Lawson JA, Brass LF (2005). Spontaneous fetal loss caused by placental thrombosis in estrogen sulfotransferase-deficient mice.. Nature medicine.

[37] Yoshinari K, Yoda N, Toriyabe T, Yamazoe Y (2010). Constitutive androstane receptor transcriptionally activates human CYP1A1 and CYP1A2 genes through a common regulatory element in the 5′-flanking region.. Biochemical pharmacology.

[38] Chang TK (2009). Activation of pregnane X receptor (PXR) and constitutive androstane receptor (CAR) by herbal medicines.. The AAPS journal.

[39] Choi JY, Lee KM, Park SK, Noh DY, Ahn SH (2005). Genetic polymorphisms of SULT1A1 and SULT1E1 and the risk and survival of breast cancer.. Cancer Epidemiol Biomarkers Prev.

[40] Rebbeck TR, Troxel AB, Wang Y, Walker AH, Panossian S (2006). Estrogen sulfation genes, hormone replacement therapy, and endometrial cancer risk.. Journal of the National Cancer Institute.

[41] Hirata H, Hinoda Y, Okayama N, Suehiro Y, Kawamoto K (2008). CYP1A1, SULT1A1, and SULT1E1 polymorphisms are risk factors for endometrial cancer susceptibility.. Cancer.

[42] He D, Wilborn TW, Falany JL, Li L, Falany CN (2008). Repression of CFTR activity in human MMNK-1 cholangiocytes induces sulfotransferase 1E1 expression in co-cultured HepG2 hepatocytes.. Biochimica et biophysica acta.

[43] Falany CN, He D, Li L, Falany JL, Wilborn TW (2009). Regulation of hepatic sulfotransferase (SULT) 1E1 expression and effects on estrogenic activity in cystic fibrosis (CF).. The Journal of steroid biochemistry and molecular biology.

[44] Kang HS, Angers M, Beak JY, Wu X, Gimble JM (2007). Gene expression profiling reveals a regulatory role for ROR alpha and ROR gamma in phase I and phase II metabolism.. Physiological genomics.

[45] Gong H, Jarzynka MJ, Cole TJ, Lee JH, Wada T (2008). Glucocorticoids antagonize estrogens by glucocorticoid receptor-mediated activation of estrogen sulfotransferase.. Cancer research.

[46] Yoshinari K, Kobayashi K, Moore R, Kawamoto T, Negishi M (2003). Identification of the nuclear receptor CAR:HSP90 complex in mouse liver and recruitment of protein phosphatase 2A in response to phenobarbital.. FEBS letters.

[47] Song WC, Moore R, McLachlan JA, Negishi M (1995). Molecular characterization of a testis-specific estrogen sulfotransferase and aberrant liver expression in obese and diabetogenic C57BL/KsJ-db/db mice.. Endocrinology.

